# Serum vitamin D in patients with mild cognitive impairment and Alzheimer's disease

**DOI:** 10.1002/brb3.936

**Published:** 2018-02-09

**Authors:** Shinji Ouma, Midori Suenaga, Funda F. Bölükbaşı Hatip, Izzettin Hatip‐Al‐Khatib, Yoshio Tsuboi, Yoichi Matsunaga

**Affiliations:** ^1^ Department of Neurology Faculty of Medicine Fukuoka University Fukuoka Japan; ^2^ Department of Medical Pharmacology Faculty of Pharmaceutical Sciences Tokushima Bunri University Tokushima Japan; ^3^ Department of Medical Pharmacology Faculty of Medicine Pamukkale University Denizli Turkey

**Keywords:** 1,25(OH)_2_D_3_, 25(OH)D_3_, Alzheimer's disease, mild cognitive impairment, mini‐mental state examination

## Abstract

**Objectives:**

To determine the relevance of Mini‐Mental State Examination (MMSE), serum 25‐hydroxyvitamin D (25(OH)D3), and 1,25(OH)2D3 concentrations to mild cognitive impairment (MCI) and various stages of Alzheimer's disease (AD).

**Materials and Methods:**

The study included 230 participants (>74 years) allocated to three main groups: 1‐healthy subjects (HS,* n* = 61), 2‐patients with MCI (*n* = 61), and 3‐ patients with Alzheimer's disease (AD) subdivided into three stages: mild (*n* = 41), moderate (*n* = 35), and severe AD (*n* = 32). The cognitive status was evaluated using MMSE. Serum 25 (OH)D3 (ng/ml) and 1,25(OH)2D3 concentrations (pg/ml) were determined by competitive radioimmunoassay.

**Results:**

MMSE scores and 25(OH)D3 were decreased in MCI and all stages of the AD in both genders. MMSE variability was due to gender in HS (11%) and to 25(OH)D3 in MCI (15%) and AD (26%). ROC analysis revealed an outstanding property of MMSE in diagnosis of MCI (AUC, 0.906; CI 95%, 0.847–0.965; sensitivity 82%; specificity, 98%) and AD (AUC, 0.997; CI 95%, 0.992–1; sensitivity, 100%; specificity, 98%). 25(OH)D3 exhibited good property in MCI (AUC, 0.765; CI 95%, 0.681–0.849; sensitivity, 90%; specificity, 54%) and an excellent property in diagnosis of AD (AUC, 0.843; CI 95%, 0.782–0.904; sensitivity, 97%; specificity, 79%). Logistic analyses revealed that, in MCI, MMSE could predict (or classify correctly) with 97.6% accuracy (Wald, 15.22, β, −0.162; *SE*, 0.554; OR = 0.115:0.039–0.341; *p* = .0001), whereas 25(OH)D3 with 80% accuracy (Wald, 41,013; β, −0.213; *SE*, 0.033; OR = 0.808: 0.757–863; *p* = .0001). 25(OH)D3 was the only significant predictor for the severe AD and contributed to MMSE variability. Age and gender were significant predictors only in the moderate AD. In patients with MCI, 25(OH)D3 and 1,25(OH)2D3 were correlated men, but in case of the AD, they were correlated in women.

**Conclusions:**

MMSE and serum 25(OH)D3 concentrations could be useful biomarkers for prediction and diagnosis of MCI and various stages of the AD. The results support the utility of vitamin D supplementation in AD therapy regimen.

## 1 INTRODUCTION

Alzheimer's disease (AD) is the most common cause of cognition impairment in elderly populations. AD is characterized by dementia with progressive loss of memory, intellectuality, disturbance of language ability, impairment in social performance, and reduced independence (i.e., the need for caregiver support in daily life). Although a definitive diagnosis of AD can only be made based on histopathological examination of brain specimens, the clinical diagnosis of AD could have a high degree of accuracy if dementia is diagnosed using a cognitive score (Creavin et al., 2016; Votruba, Persad, & Giordani, 2016). In addition to age and gender, the Mini‐Mental State Examination (MMSE) has been regarded as a useful instrument for evaluating the cognitive state of patients (Folstein, Folstein, & McHugh, 1975) and used as a predictor of AD (Musicco et al., 2009). Mild cognitive impairment (MCI) is subclinical complaint of memory function in elderly people. It has been reported that 10%–20% of individuals over the age of 65 years suffer from MCI (Petersen, 2011), with high potential of converting to AD (Devanand et al.,^2008^; Ganguli et al., 2011; Petersen et al., 2001; Ritchie & Touchon, 2000).

In addition to its known significance in bone and calcium homeostasis, vitamin D improves protein homeostasis and slows aging (Mark et al., 2016). The enzymes involved in conversion of 25(OH)D_3_ to 1,25(OH)_2_D_3_ are all present in the brain (Harms, Burne, Eyles, & McGrath, 2011). There is a reciprocal relationship between vitamin D and AD. It has been reported that 25(OH)D_3_ is reduced in late‐onset AD, and vitamin D deficiency is regarded as a risk factor for ApoEε4 noncarrier patients with AD (Dursun et al., 2016). On the other hand, supplementation with vitamin D derivatives decreases the risk of AD (Dean, Bellgrove, & Hall, 2011). It has recently been found that vitamin D receptors are colocalized with amyloid precursor protein on the neuronal plasma membrane (Dursun & Gezen‐Ak, 2017). Amyloid β (Aβ), the pathological hallmark of AD, degrades vitamin D receptor (Dursun, Gezen‐Ak, & Yilmazer, 2010). Vitamin D decreases the burden of major pathological aggregates in AD, including Aβ plaques and hyperphosphorylated tau protein (Durk, Han, & Chow, 2014; Yu et al., 2011) and augments activity of memantine in AD (Lemire, Brangier, Beaudenon, Duval, & Annweiler, 2016). Moreover, many reports suggest a relationship between vitamin D deficiency with MCI (Annweiler et al., 2012; Yin, Fan, Lin, Xu, & Zhang, 2015) and AD (Annweiler et al., 2010, 2011; Landel, Annweiler, Millet, Morello, & Féron, 2016; Nissou et al., 2014; Schlögl & Holick, 2014).

There are several measures that could distinguish AD from control subjects. These include decreased metabolism of fluorodeoxyglucose (Silverman et al., 2001), increased uptake of amyloid (Small et al., 2006), elevated levels of tau or its phosphorylated form, and decreased amyloid β42 in CSF (Hansson et al., 2006; Querfurth & LaFerla, 2010; Sunderland et al., 2003). However, these approaches are either invasive or very expensive. Therefore, there is still a need for developing diagnosis as well as treatment approaches to diseases characterized by dementia. Also, as to our knowledge, no study of possible use of both 25(OH)D_3_ and 1,25(OH)_2_D_3_, separately or in combination with MMSE, as predictors in diagnosis and prediction of MCI and various stages (mild, moderate, and severe) of AD. Accordingly, this study was conducted to evaluate utility of MMSE, serum 25(OH)D_3,_ and 1,25(OH)_2_D_3_ concentrations in prediction and diagnosis patients with MCI and the various stages of AD.

## 2 MATERIALS AND METHODS

### 2.1 Participants

A total of 230 individuals from Fukuoka University Hospital were included in this study. The participants were allocated to three groups: I‐Healthy subjects (HS), II‐patients with MCI and III‐ patients with AD main group classified, according to disease severity, into three stages defined as 1‐mild AD, 2‐moderate AD, and 3‐severe AD. Diagnosis of MCI was performed according to Petersen's criteria (Petersen, 2011; Petersen et al., 2001; Ritchie & Touchon, 2000). The severity of cognitive impairment in patients with AD was evaluated using MMSE scores: mild AD (27 ≥ MMSE > 20), moderate AD (20 ≥ MMSE > 10), and severe AD (10 > MMSE) (Feldman, Van Baelen, Kavanagh, & Torfs, 2005; O'Bryant, Humphreys, & Smith, 2008). Each participant was clinically evaluated by set of tests that included questionnaire and a proxy interview, assessment of past and present illness, neurological and physical examinations, blood chemistry, and neuroimaging with computed tomography and/or magnetic resonance imaging. Some participants in the HS group had hypertension (eight of 33; 24%) and/or hypercholesterolemia (four of 33; 12%), regarded above the baseline blood pressure (systolic 139/diastolic 89 mmHg) and cholesterol (219 mg/dl). All groups were gender‐balanced except there were two times as many women as men in the moderate and severe AD groups. Moreover, the groups were also age‐matched except the difference between women moderate AD compared to women HS (*p *=* *.23) and men HS (*p = *.013). The respective ages (year) of women and men were as follows: HS (74.5 ± 6.3; 74.4 ± 8.7), MCI (75.5 ± 6.8, 77.7 ± 11.2), mild AD (74.8 ± 8.1; 78.3 ± 6.3), moderate AD (82.2 ± 5.1; 76.9 ± 7.6), and severe AD (77.7 ± 8.7; 76.5 ± 9.1). A difference was detected only between moderate AD women and HS women (*p *=* *0.031), and between moderate AD women and moderate AD men (*p *=* *0.016). This difference is consequent to grouping according to the clinical classification to MCI or AD. All participants were free of hepatic and renal disorders. The ethical permission for this study was obtained from the ethical committee of Fukuoka University Hospital. The study was performed in accordance with the ethical standards of the 1964 Helsinki Declaration. Written informed consent was obtained from all participants or their relatives prior to their participation in the study. We excluded participants with any present or earlier history of vitamin D supplementation.

### 2.2 Samples preparations and analyses

Peripheral blood was collected from each participant and centrifuged at 400 x *g* for 20 min. The sera obtained were stored at −80°C until use. Total serum concentrations of 25(OH)D_3_ and 1,25(OH)_2_D_3_ were determined by competitive radioimmunoassay using two respective antibodies. A 25‐OH vitamin D ^125^I RIA Kit (DiaSorin Inc. MN, USA) was used to assay 25(OH)D_3_. Briefly, after pretreatment of the samples with acetonitrile 300 to remove proteins, the sample extracts containing 25(OH)D_3_ were incubated with ^125^I‐25(OH)D_3_ and sheep anti‐25(OH)D_3_ antibody for 90 min at room temperature. Cellulose‐conjugated anti‐sheep IgG antibody was added to the precipitated reactive complex and free ^125^I‐25(OH)D_3_ was removed by centrifugation. The radioactivity in each precipitate was assayed using a γ‐counter (ARC‐950, Hitachi‐Aloka Medical Ltd, Japan), and concentrations were determined according to a standard curve. A 1,25(OH)_2_D_3_ RIA Kit (Immunodiagnostic Systems Ltd, Boldon, England) was used to assay 1,25(OH)_2_D_3_. The principle of this assay system was the same as that above except a column technique was also employed to remove lipids during sample pretreatment.

### 2.3 Statistical analyses

ANOVA one‐way was conducted on the variables (age, MMSE, 25(OH)D3 and 1,25(OH)2D3) between groups (HS, MCI, mild AD, moderate AD and severe D), with gender as covariate, to detect the following: 1‐the main effect, differences between the variables of the groups, 2‐The groups within each gender, and each gender's variable between groups were compared to evaluate the effects of gender. Homogeneity was verified. Tukey's multiple comparisons post hoc test was applied whenever ANOVA detected significant differences. Bivariate correlations among the variables were evaluated by Pearson's correlation coefficient. As MMSE could be seen as both risk factor and outcome of the disease, a linear regression analysis was also conducted to determine the regression coefficients, statistical significance of regression model (*t* value), and proportion of MMSE (dependent) contributed by independent variables (age, gender, 25(OH)D3, and 1,25(OH)_2_D3) derived from the multiple correlation coefficient (Adjusted *R* ^2^).

The predictors were also tested with univariate logistic regression analyses to assess the contribution of each predictor alone to each group. Then, multivariate‐forward selection analyses were conducted to assess the contribution of the predictors in combination to increase the statistical power and account for the individual differences in prediction. Variables which had a *p* value of >.05 were excluded. The followings were calculated: β: logistic regression coefficient describes the size and direction of the relationship between a predictor and the disease (predictive value). Positive predictive value is the probability that a subject classified as a patient by the test belongs in the patient group becomes more likely as the predictor increases. Negative predictive value is the probability that a subject classified as a nonpatient by the test belongs in the nonpatient group. It also indicates the inverse relationship between the predictor and the disease (decreased predictor means increased disease odd). Odd ratio (OR): the ratio of the odds, calculated as the exponent of β. OR is the measurement of likelihood and indicates that when the predictor is raised by one unit the odds ratio of the outcome increase by a factor equal to the OR value, that is, the odds of participants in the dependent variable (patients) increase by a factor equivalent to OR value with 95% confidence interval (CI). Correct classification, CC (accuracy rate (%) of the predictor to diagnose or distinguish two compared variables), and Wald value (significance of predictor contribution) were also measured.

Receiver operating characteristic (ROC) analysis provides useful information regarding the ability of a predictor to classify subjects into the relevant groups, and to compare the performance of more than one predictor. ROC was conducted to calculate area under the ROC curve (AUC), sensitivity, and specificity. Cutoff values at which optimal balance of sensitivity and specificity can be obtained were derived according to Youden Index. Sensitivity (with optimal 95% confidence interval) is the probability that a test result will be positive when the disease is present (true positive rate—the probability that a patient will be accurately classified by the test). Specificity (with optimal 95% confidence interval) is probability that a test result will be negative when the disease is not present (true negative rate—the probability that a nonpatient will be accurately classified by the test). The AUC is a measure of the efficacy of the test. The AUC values are typically interpreted as chance (0.0–0.4), poor (0.5–0.6), weak (0.6–0.7), good or acceptable (0.7–0.8), excellent or great (0.8–0.9), and perfect or outstanding (0.9–1.0). The higher the AUC, the more true positive is the result. The positive (LR+) and negative (LR−) likelihood ratios are probabilities of respective positive and negative test results. They can be derived from sensitivity and specificity: LR+ = (Sensitivity or True positive/1 − Specificity or False positive); LR− = (1 − Sensitivity or False negative/Specificity or True negative).

**Figure 1:**
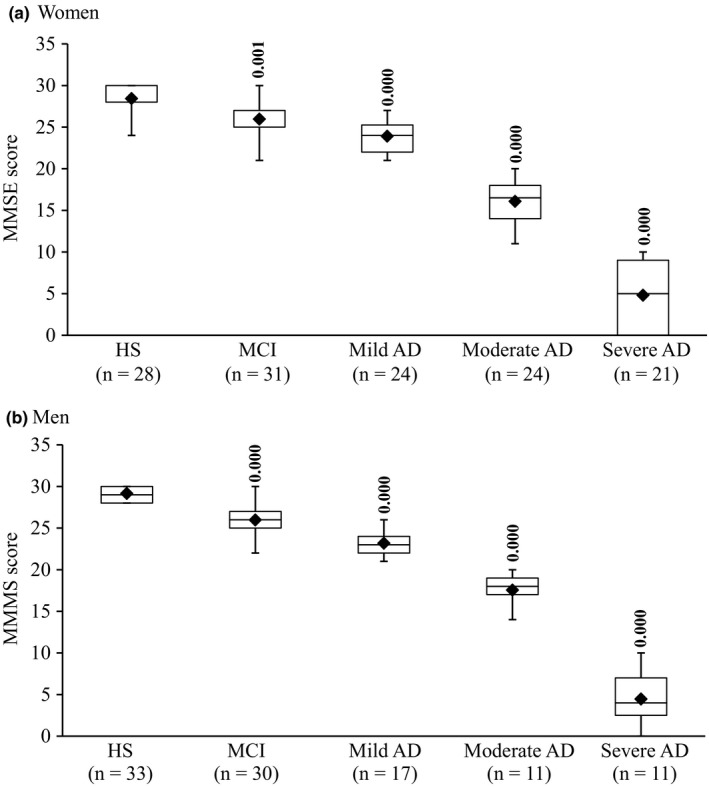
Box plot of MMSE scores in women (a) and men (b). The lower and upper sides of the boxes indicate the 25th and 75th percentiles. The horizontal lines and black diamonds inside the boxes indicate the median and means, respectively. Shown are also the lower and upper whiskers that indicate the minimum and maximum values, respectively. In women HS, the upper horizontal bar outside the box with the whisker and the median line inside the box have not appeared because the maximum and 75th percentiles, and median and 25th percentiles are at the same level. In women MCI, the median line has not appeared because 75th percentiles and median are at the same level. For the same above‐mentioned reasons, the upper and lower horizontal bars outside the box with the whiskers have not appeared in men HS. The levels of statistically significant differences are indicated over each point. AD and MCI are compared with HS

**Figure 2:**
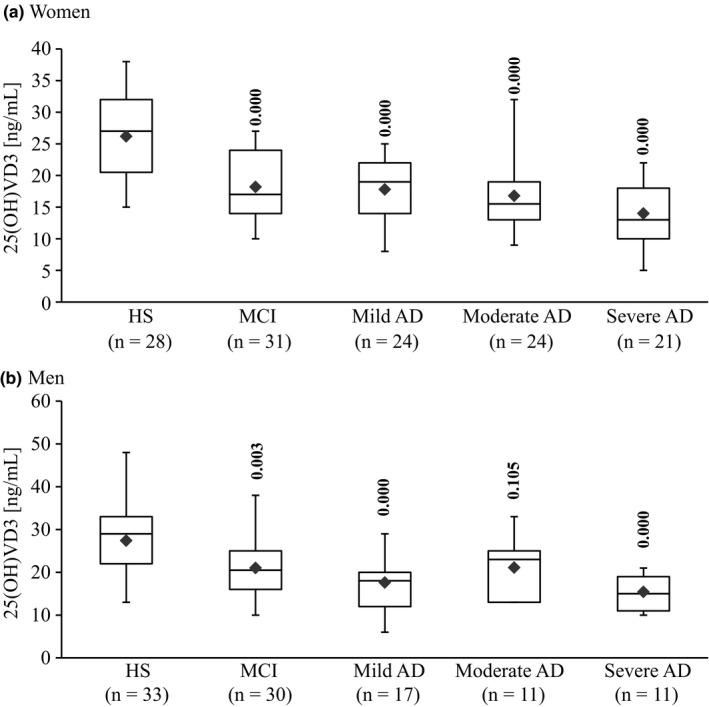
Box plot of serum concentrations of 25(OH)D_3_ in women (a) and men (b) HS, MCI and AD. The lower and upper sides of the boxes indicate the 25th and 75th percentiles. The horizontal lines and black diamonds inside the boxes indicate the median and means, respectively. Shown are also the lower and upper whiskers that indicate the minimum and maximum values, respectively. The levels of statistically significant differences are indicated over each point. Alzheimer's disease (AD) and mild cognitive impairment (MCI) compared with healthy subjects (HS)

**Figure 3:**
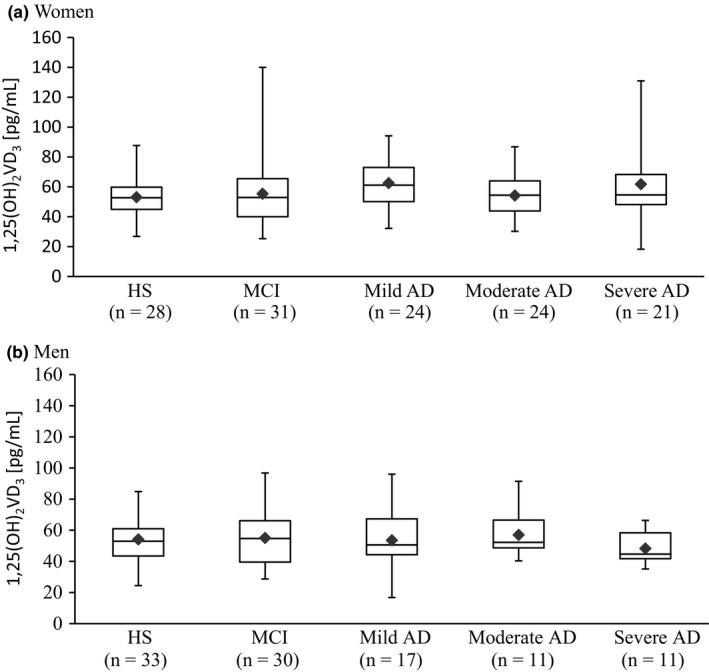
Box plot of serum concentrations of 1,25(OH)D_3_ in women (a) and men (b). The lower and upper sides of the boxes indicate the 25th and 75th percentiles. The horizontal lines and black diamonds inside the boxes indicate the median and means, respectively. Shown are also the lower and upper whiskers that indicate the minimum and maximum values, respectively. No significant differences were detected among healthy subjects (HS), patients with mild cognitive impairment (MCI), and patients with Alzheimer's disease (AD)

The criterion for statistical significance was *p* < .05. The values are presented as the mean ± standard deviation in Figures 1, 2, 3, and as the standard error in the tables. The data were analyzed, using IBM SPSS Statistics version 23.

## 3 RESULTS

### 3.1 MMSE scores in patients with MCI and AD

The MMSE scores of HS women and men were 28.0 ± 1.9, 29.1 ± 0.9, respectively. Figure 1 shows that MMSE scores were decreased in MCI and AD. A significant difference for the main effect, between groups, was detected for MMSE (*F*(4,225) = 722.076; *p *=* *.000). There was no difference in the MMSE values between women (26.0 ± 2.4) and men (26.0 ± 1.8) with MCI. The MMSE scores were decreased in mild AD (women 23.9 ± 2.0, men 23.2 ± 1.6), moderate AD (women 16.1 ± 2.5, men 17.5 ± 2.0), and severe AD (women 4.6 ± 4.1, men 4.5 ± 3.4). The decrease in MMSE scores in AD was more than that observed in MCI in both genders (*p *=* *.000 for moderate and severe AD vs. MCI) except in mild AD (women, *p *=* *.030; men, *p *=* *.002 vs. MCI). In addition, significant differences (*p *=* *.000) were detected among the various stages of AD in women and men analyzed separately. However, no significant gender‐dependent difference was detected for the same stage of AD between women and men when compared to each other.

### 3.2 Serum 25(OH)D_3_ concentrations in patients with MCI and AD

In HS, the mean serum concentrations of 25(OH)D_3_ were 26.18 ± 7.18 ng/ml and 27.42 ± 8.05 ng/ml in women and men participants, respectively. A significant (*F*(4,225) = 25.869, *p *=* *.000) main effect of 25(OH)D_3_ was obtained in MCI and AD. Figure 2 shows that concentrations of 25(OH)D_3_ in patients with MCI were lower than HS in both women (18.23 ± 5.11 ng/ml; *p *=* *.000) and men (21.03 ± 6.99 ng/ml; *p *=* *.003). However, the concentrations of 25(OH)D_3_ in MCI were not different from AD in both genders.

Figure 2a shows that in AD women patients, 25(OH)D_3_ concentrations in mild AD (17.75 ± 5.30 ng/ml), moderate AD (16.79 ± 5.32 ng/ml), and severe AD (13.95 ± 5.08 ng/ml) were significantly lower than HS (*p *=* *.000). On the other hand, it can be seen from Figure 2b that in the men patients, the concentrations of 25(OH)D_3_ were significantly (*p *=* *.000) lower than HS in mild AD (17.59 ± 6.95 ng/ml) and severe AD (15.36 ± 4.08 ng/ml). However, no significant (*p *=* *.105) difference was detected between HS and the moderate AD (21.09 ± 6.32 ng/ml). No significant difference was detected among the AD stages for the same gender, or between the genders in each group.

### 3.3 Serum 1,25(OH)_2_D_3_ concentrations in patients with MCI and AD

In HS, the mean serum concentrations of 1,25(OH***)***_2_D_3_ were 53.05 ± 13.04 pg/ml and 54.12 ± 14.34 pg/ml in women and men participants, respectively. Figure 3a shows that, in women, the concentrations of 1,25(OH)_2_D_3_ were 55.38 ± 22.85 pg/ml (MCI), 62.53 ± 17.03 pg/ml (mild AD), 54.22 ± 13.71 pg/ml (moderate AD), and 61.84 ± 26.45 pg/ml (severe AD). Moreover, Figure 3b shows that, in men, the concentrations of 1,25(OH)_2_D_3_ were 55.01 ± 18.00 pg/ml (MCI), 53.52 ± 19.20 pg/ml (mild AD), 57.02 ± 15.03 pg/ml (moderate AD), and 48.25 ± 9.55 pg/ml (severe AD). No significant difference among the groups was detected (*F*(4,225) = 0.583, *p *=* *.676).

### 3.4 Correlations among MMSE, 25(OH)D_3,_ and 1,25(OH)_2_D_3_

**Figure 4:**
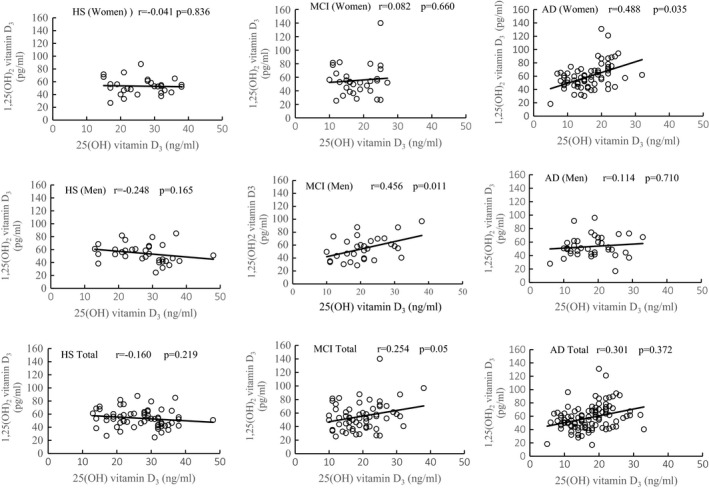
Correlations between serum 25(OH)D_3_ and 1,25(OH)_2_D_3_ concentrations in women and men healthy subjects (HS), mild cognitive impairment (MCI), and Alzheimer's disease (AD). Shown are also the total correlation values for both genders in combination (total) in each group

Table 1 and Figure 4 show that no correlation was evident in HS. On the other hand, in MCI, the largest and significant correlation was detected in men between 25(OH)D_3_ and 1,25(OH)_2_D_3_ (*r* = .456, *p *=* *.011) in addition to the correlation between 25(OH)D_3_ and MMSE (*r* = .330, *p *=* *.022), and 1,25(OH) _2_D_3_ (*r* = −.356, *p *=* *.048). The total correlation between 25(OH)D_3_ and 1,25(OH)_2_D3 in both genders was significant (*r* = .254, *p *=* *.05). No significant correlation was observed in women with MCI.

**Table 1:** Correlations among MMSE, 25 (OH)D3 and. 1,25(OH)2 D3 in healthy subjects (HS) and patients with mild cognitive impairment (MCI) and Alzheimer's diseases (AD)

Women			25 (OH)D3	1,25 (OH)2 D3	Men			25 (OH)D3	1,25 (OH)2 D3
HS (*n* = 28)	MMSE	*r*	.122	.147	HS (*n* = 33)	MMSE	*r*	−.139	−.134
		*p*	.535	.258			*p*	.441	.457
	25(OH)D3	*r*	1	−.041		25(OH)D3	*r*	1	−.248
		*p*	—	.836			*p*	—	.165
MCI (*n* = 31)	MMSE	*r*	−.016	−.288	MCI (*n* = 30)	MMSE	*r*	.330	−.365
		*p*	.932	.115			*p*	.022^a^	.048^a^
	25(OH)D3	*r*	1	.082		25(OH)D3	*r*	1	.456
		*p*	—	.66			*p*	—	.011^a^
Mild AD (*n* = 24)	MMSE	*r*	−.086	−.099	Mild AD (*n* = 17)	MMSE	*r*	.052	−.275
		*p*	.689	.645			*p*	.842	.285
	25(OH)D3	*r*	1	.487		25(OH)D3	*r*	1	.163
		*p*	—	.016^a^			*p*	—	.531
Moderate AD (*n* = 24)	MMSE	*r*	−.326	.148	Moderate AD (*n* = 11)	MMSE	*r*	−.415	−.280
		*p*	.048^a^	.49			*p*	.205	.404
	25(OH)D3	*r*	1	.357		25(OH)D3	*r*	1	.156
		*p*	—	.087			*p*	—	.647
Severe AD (*n* = 21)	MMSE	*r*	−.331	.239	Severe AD (*n* = 11)	MMSE	*r*	.138	.056
		*p*	.023^a^	.297			*p*	.687	.87
	25(OH)D3	*r*	1	.62		25(OH)D3	*r*	1	.024
		*p*	—	.003^a^			*p*	—	.944

The correlation coefficient magnitude (*r*) is determined according to Pearson's correlation: small (*r* = .1), medium (*r* = .3), and large (*r *= >.5).

a Significant correlation.

In patients with AD, significant correlations were detected only in women. MMSE was correlated only with 25(OH)D_3_ in moderate AD (*r* = −.326, *p *=* *.048) and severe AD (*r* = −.331, *p *=* *.023), indicating to a parallel decrease of MMSE with 25(OH)D_3_, but not 1,25(OH)_2_D_3_. On the other hand, the largest correlation was detected between 25(OH)D_3_ and 1,25(OH)_2_D_3_ in severe AD (*r* = .62; *p *=* *.003) followed by mild AD (*r* = .487, *p *=* *.016). There was no significant correlation between 25(OH)D_3_ and 1,25(OH)_2_D_3_ in moderate AD (*r* = .357, *p *=* *.087). The correlation of 25(OH)D_3_ and 1,25(OH)_2_D_3_ values of all stages together was significant in women (*r* = .488, *p *=* *.016) but not in men (*r* = .114, *p *=* *.710). The total correlation between 25(OH)D_3_ and 1,25(OH)_2_D_3_ for both genders was not significant (*r* = .301, *p *=* *.372).

The linear regression established that only gender could significantly predict MMSE (*B* = 0.325, *t* = 2.638, *p *=* *.011) and account for 11% (adjusted *R* ^2^) of MMSE variability in HS. On the other hand, although both 25(OH)D_3_ and 1,25(OH)_2_D_3_ contributed to MMSE variability in MCI, 1,25(OH) _2_D_3_ accounted for only 3% of the variability (*p *=* *.075), while 25(OH)D_3_ significantly (*p *=* *.0001) contributed (16%) to MMSE variability. On the other hand, in AD, only 25(OH)D_3_ could significantly (*p *=* *.000) affect MMSE and accounts for 26% of MMSE variability (Table 2).

**Table 2:** Linear regression of gender, 25(OH)D3 and 1,25(OH)2D3 with MMSE in healthy subjects (HS) and patients with mild cognitive impairment (MCI) and Alzheimer's diseases (AD)

Group	Model	Predictor	USC B	*SE*	SC Beta	*t* value	Sig.	95% CI for B		*R* ^2^	Adjusted *R* ^2^
								Lower bound	Upper bound		
HS	Univariate	25(OH)D3	0.004	0.019	0.024	0.186	0.853	0.035	0.042	.001	−.016
	Univariate	1,25(OH)2D3	0.012	0.011	0.147	1.142	0.258	0.009	0.033	.022	.005
	Multivariate	Gender	0.723	0.274	0.325	2.638	0.011*	0.175	1.271	.106	.09
MCI	Univariate	25(OH)D3	0.098	0.024	0.346	4.041	0.0001*	0.05	0.145	.12	.112
	Univariate	1,25(OH)2D3	‐0.023	0.011	0.177	1.973	0.075	0.045	0	.031	.023
	Multivariate	25(OH)D3	0.098	0.024	0.346	4,041	0.0001*	0.05	0.145	.12	.112
AD	Univariate	25(OH)D3	0.572	0.076	0.506	7.574	0.0001*	0.423	0.722	.256	.251
	Univariate	1,25(OH)2D3	−0.014	0.042	0.026	0.34	0.735	−0.097	0.068	.001	.005
	Multivariate	25(OH)D3	0.572	0.076	0.506	7.574	0.0001*	0.423	0.722	.256	.251

USC B: unstandardized regression coefficient; SC Beta: standardized coefficient; *R* ^2^: squared multiple correlation coefficient; *SE*: standard errors of the regression coefficients; Sig: two‐sided observed significance levels (*p*) for the *t* statistics. CI: confidence interval; *Significant *p* values. The predictors 25(OH)D3, 1,25(OH)2D3 and gender (independent variable) were used to predict MMSE (dependent variable).

### 3.5 Receiver operating characteristic (ROC) analysis

Table 3 shows that MMSE displayed an excellent–perfect diagnostic power and differentiating MCI from HS, various AD stages among themselves and each stage from HS. However, MMSE exhibited the same or close sensitivity and specificity values. The highest cutoff value was obtained in MCI and AD when evaluated against HS. Moreover, higher LR+ and lower LR− for MMSE were observed in MCI and mild AD combined with HS (data not shown). On the other hand, an excellent–perfect differential diagnostic power was detected for 25(OH)D_3_ when either MCI or AD subgroups were evaluated against HS. However, 25(OH)D_3_ exhibited poor–weak power when the differential diagnosis was analyzed among the stages of AD. Moreover, high sensitivity and low specificity were obtained for 25(OH)D_3_ especially in MCI and AD when evaluated against HS. The cutoff value of 25(OH)D_3_ was higher than MMSE, especially in patients with AD whether evaluated against HS or other groups of AD. Moreover, higher (10 times) LR+ than LR− was observed for 25(OH)D3 (data not shown).

**Table 3:** Bivariate receiver operating characteristic (ROC) analysis of the study groups

		AUC	*SE*	Sig	CI (95%)		Cut‐ off^a^	Sensitivity	Specificity
					Lower Bound	Upper Bound			
MCI vs. HS	MMSE	0.906	0.03	0.0001^b^	0.847	0.965	27.5	0.82	0.984
	25 (OH)D3	0.765	0.04	0.0001^b^	0.681	0.849	27.5	0.902	0.541
	1,25(OH)2 D3	0.5	0.05	1.000	0.396	0.604	—	—	—
Mild AD vs. HS	MMSE	0.993	0.01	0.0001^b^	0.979	1	27.5	1	0.984
	25 (OH)D3	0.815	0.04	0.0001^b^	0.736	0.895	25.5	0.927	0.574
	1,25(OH)2 D3	0.41	0.06	0.124	0.293	0.527	—	—	—
Moderate AD vs. HS	MMSE	1	0	0.0001^b^	1	1	22	1	1
	25 (OH)D3	0.812	0.04	0.0001^b^	0.725	0.899	27.5	0.943	0.541
	1,25(OH)2 D3	0.472	0.06	0.645	0.351	0.593	—	—	—
Severe AD vs. HS	MMSE	1	0	0.0001^b^	1	1	17	1	1
	25 (OH)D3	0.911	0.03	0.0001^b^	0.857	0.966	20.5	0.906	0.787
	1,25(OH)2 D3	0.493	0.07	0.913	0.367	0.62	—	—	—
Mild AD vs. Moderate AD	MMSE	1	0	0.0001^b^	1	1	20.5	1	1
	25 (OH)D3	0.494	0.07	0.929	0.363	0.626	—	—	—
	1,25(OH)2 D3	0.566	0.07	0.327	0.436	0.695	—	—	—
Severe AD vs. Mild AD	MMSE	1	0	0.0001^b^	0	0	15.5	1	1
	25 (OH)D3	0.661	0.06	0.019^b^	0.538	0.785	21.5	0.341	0.969
	1,25(OH)2 D3	0.566	0.07	0.333	0.433	0.7	—	—	—
Severe AD vs. Moderate AD	MMSE	1	0	0.0001^b^	1	1	10.5	1	1
	25 (OH)D3	0.675	0.07	0.014^b^	0.546	0.804	21.5	0.314	0.969
	1,25(OH)2 D3	0.515	0.07	0.836	0.374	0.655	—	—	—
Total AD vs. HS	MMSE	0.997	0.003	0.0001^b^	0.992	1	27.5	1	0.984
	25 (OH)D3	0.843	0.03	0.0001^b^	0.782	0.904	20.5	0.741	0.787
	1,25(OH)2 D3	0.455	0.05	0.327	0.366	0.543	—	—	—

AUC, area under the ROC curve; CI, confidence interval; *SE*, standard error of AUC.

a Cutoff values at which optimal balance of sensitivity and specificity can be obtained according to Youden's index; Youden's Index can be calculated as the sum of sensitivity plus specificity minus 1 for all possible cutoff points.

b Sig, significance of AUC.

### 3.6 Univariate and multivariate logistic regression analyses

The prediction values were evaluated for each of MCI and AD groups against HS. Univariate analysis of each predictor alone (Table 4) shows that significant (*p *=* *.0001) prediction by age (β = +0.133; Wald = 12.12; OR = 1.143; CC = 71%; *p *=* *.0001) and gender (β = −0.944; Wald = 4.49; OR = 0.389; CC = 64%; *p *=* *.034) was obtained in moderate AD. These results indicate that for each one unit increase of age (1 year), the odds of disease risk increases by 1.143 (53% probability). In case of gender, women were considered as the reference. In other words, the negative value of β indicates that women have 2.6 (1/0.389 = 2.6) times the risk of the disease than men. Moreover, it can also be seen from Table 4 that a significant (*p* = .0001) prediction by MMSE was observed in MCI (β = −1.324, Wald = 27.86, OR = 0.266) and mild AD (β = −2.162, Wald = 15.22, OR = 0.115). These results indicate that each unit decrease of MMSE reflects 21% and 13% probability increase in the prediction odds of MCI and mild AD, respectively. Table 4 also shows that the higher OR and Wald values were detected for 25(OH)D3 in MCI (β = −0.146, Wald = 22.044, OR = 0.864, 46% probability), mild AD (β = −0.188, Wald = 22.744, OR = 0.829, 45% probability), moderate AD (β = −0.178, Wald = 20.04, OR = 0.837, 46% probability), and severe AD (β = −0.316, Wald = 20.821, OR = 0.729, 42%). These results indicate that each unit decrease of 25(OH)D_3_ reflects an increase in the prediction OR (by about 80%) and probability (>40%) rate indicated for each of MCI and AD groups. Multivariate analysis of all predictors combined within each group revealed that only MMSE and 25(OH)D_3_ displayed significant prediction of MCI.

**Table 4:** Logistic regression analyses for the contribution of the individual and combined predictors

	β	*SE*	Wald	Sig.	OR	OR 95% CI		CC%
						Lower	Upper	
MCI‐HS								
A‐Univariate								
Age	0.033	0.023	1.976	0.16	1.034	0.987	1.082	57.4
Gender	−0.197	0.363	0.295	0.587	0.821	0.403	1.672	52.5
MMSE	−1.324	0.251	27.858	0.0001*	0.266	0.163	0.435	90.2
25 (OH)D3	−0.146	0.031	22.044	0.0001*	0.864	0.813	0.918	70.2
1,25(OH)2 D3	0.005	0.011	0.251	0.616	1.005	0.985	1.026	50.8
B‐Multivariate								
MMSE	−1.445	0.295	24.041	0.0001*	0.236	0.132	0.42	88.5
25 (OH)D3	−0.168	0.047	12.644	0.0001*	0.846	0.771	0.927	
Mild AD‐HS								
A‐Univariate								
Age	0.033	0.028	1.41	0.235	1.033	0.979	1.091	61.8
Gender	−0.509	0.408	1.557	0.212	0.601	0.27	1.337	59.8
MMSE	−2.162	0.554	15.22	0.0001*	0.115	0.039	0.341	96.1
25 (OH)D3	−0.188	0.039	22.744	0.0001*	0.829	0.767	0.895	71.6
1,25(OH)2 D3	0.021	0.013	2.578	0.108	1.021	0.995	1.048	61.8
Moderate AD‐HS								
A‐Univariate								
Age	0.133	0.038	12.119	0.0001*	1.143	1.06	1.231	70.8
Gender	−0.944	0.446	4.492	0.034*	0.389	0.162	0.931	63.5
MMSE	−0.053	846.038	0	0.992	0	0	0	100
25 (OH)D3	−0.178	0.04	20.04	0.0001*	0.837	0.774	0.905	75
1,25(OH)2 D3	0.008	0.016	0.256	0.613	1.008	0.978	1.039	63.5
Severe AD‐HS								
A‐Univariate								
Age	0.048	0.029	2.628	0.105	1.049	0.99	1.111	66.7
Gender	−0.811	0.452	3.215	0.073	0.444	0.183	1.078	65.6
MMSE	−0.456	397.131	0	0.995	0.086	0	0	100
25 (OH)D3	−0.316	0.069	20.821	0.0001*	0.729	0.636	0.835	78.5
1,25(OH)2 D3	0.012	0.013	0.86	0.354	1.012	0.987	1.037	67.7
All participants								
A‐Univariate								
Age	0.059	0.021	7.502	0.006*	1.06	1.017	1.106	62.1
Gender	−0.735	0.326	5.087	0.024*	0.48	0.253	0.908	63.9
MMSE	−0.162	0.554	15.22	0.0001*	0.115	0.039	0.341	97.6
25(OH)D3	−0.213	0.033	41.013	0.0001*	0.808	0.757	0.863	79.9
1,25(OH)2 D3	0.013	0.01	1.635	0.201	1.013	0.993	1.033	63.9
B‐Multivariate								
MMSE	−2.83	1.022	7.662	0.006*	0.059	0.008	0.438	98.2
25(OH)D3	−0.207	0.173	2.262	0.0018*	0.813	0.579	1.141	

β: Logistic regression coefficient; CC%: correct classification %; CI: confidence interval; OR: Odd ratio (Exponent of β), Sig: Logistic Regression *p* values for Wald. Only significant variables are retained in the B. Disease groups were tested against HS. Predictor power was evaluated in MCI and AD tested against HS.

Table 4 shows that univariate analysis of all groups collectively revealed the significant contribution of all the examined predictors. The maximum Wald (41.013) and CC% (97.6%) were exhibited by 25(OH)D_3_ and MMSE, respectively. Again, multivariate analysis showed that only combined MMSE and 25(OH)D_3_ retained their significant prediction and exhibited 98% accuracy in distinguishing and predicting the diseases.

## 4 DISCUSSION

The present results show that MMSE and 25(OH)D_3_ (but not 1,25(OH)_2_D_3_) were decreased in MCI and various stages of AD. Although MMSE is one of the most widely used tools in the evaluation of cognitive status, there is still a debate about its diagnostic accuracy. Some studies have reported that MMSE lacks diagnostic specificity and has limited diagnostic accuracy, particularly for distinguishing between normal cognition and MCI, and MCI from demential patients with AD (Chapman et al., 2016) and in measuring the progression of Alzheimer's disease (Clark et al., 1999). On the other hand, MMSE has been regarded as a good first step in the evaluation of cognitive status and effectively separating those with mild AD from normal aging and MCI (Benson, Slavin, Tran, Petrella, & Doraiswamy, 2005). It has high test–retest reliability values, ranging from 0.79 to 0.99 (Folstein et al., 1975). MMSE had also been reported to predict converters to AD (Devanand et al., 2008; Palmqvist et al., 2012). The present study highlights the value of MMSE and 25(OH)D_3_ in the differential diagnosis and prediction of MCI, mild AD, moderate AD, and severe AD at a sensitivity rate >80. The differences among the results reported for MMSE could be attributed to the analyses approach such as selection of the cutoff values, and the patients’ cultural, education, and demographic specificities. It is also noteworthy to mention that MMSE could be influenced by changes that could accompany dementia. A low level of the MMSE score is associated with low plasma phosphate (Haglin & Backman, 2016).

Vitamin D3 is produced in the skin from 7‐dehydrocholesterol under the influence of UV light. Vitamin D is metabolized first to 25(OH)D_3_ in the liver, then undergoes 1α‐ hydroxylation to the hormonal form 1,25(OH)_2_D_3_ in the kidney (Bikle, 2014). The relation of 25(OH)D_3_ and 1,25(OH)_2_D_3_ is farther than that between a substrate and its product. 25(OH)D_3_ and 1,25(OH)2D_3_ are synthesized, regulated, and changed differently in variable diseases. While 25(OH)D_3_ is mainly synthesized by CYP2R1 (endoplasmic reticulum), CYP27B1 (mitochondrial) is the main enzyme involved in the synthesis of 1,25(OH)_2_D_3_. The independence of 1,25(OH)_2_D_3_ concentration from that of its precursor (25(OH)D_3_) is expected and could be attributed to their different kinetics and regulation. While 25(OH)D_3_ (prehormone) concentration is increased by a high dose of vitamin D, plasma levels of 1,25(OH)_2_D_3_ (adaptive hormone) appeared to fall with increasing doses of vitamin D, presumably because the 1‐hydroxylase system is shut down (Jones, Strugnell, & DeLuca, 1998). 1,25(OH)_2_D_3_ inhibits its own synthesis and that of its precursor 25(OH)D_3_ (Bell, Shaw, & Turner, 1984). It has been reported that 1,25(OH)_2_D_3_ kinetics do not change by aging in healthy men and women (Eastell et al., 1991; Halloran, Portale, Lonergan, & Morris, 1990). The production of 1,25(OH)2D3 could take place extrarenally and regulated endocrinologically. It has been reported that 1,25(OH)_2_D_3_ is increased by parathyroid hormone (Eastell et al., 1991) and cytokines, including TNF (Bikle & Vitamin, 2014). However, serum 25(OH)D3 is negatively correlated with TNFα, IL‐1β or IL‐6 levels in healthy subjects and patients with MCI, but positively with late‐onset AD (Dursun et al., 2016). As TNF is increased in AD (Gezen‐Ak et al., 2013), it is possible that the increased TNFα is responsible for the decreased 25(OH)D_3_. These mechanisms could play a role in maintaining 1,25(OH)_2_D_3_ level against reduced 25(OH)D_3_.

The serum vitamin D level is associated with its activity in the brain. Serum 25(OH)D_3_ concentration is correlated with regional cerebral blood flow (Farid et al., 2012), brain volume and gray matter thickness (Brouwer‐Brolsma et al., 2015; Buell et al., 2010; Hooshmand, Lökk, & Solomon, 2014) and clearance of aggregated Aβ in the AD brain (Durk et al., 2014; Masoumi, Goldenson, & Ghirmai, 2009; Yu et al., 2011). On the other hand, low serum 25(OH)D_3_ is associated with neuronal damages (Gezen‐AK, Yilmazer, & Dursun, 2014), MCI (Annweiler et al., 2012), multidomain MCI (Yin et al., 2015) and AD (Annweiler et al., 2015; Balion, Griffith, & Strifler, 2012; Chei et al., 2014; Littlejohns, Henley, & Lang, 2014). The present results showed that 25(OH)D_3_ is involved in the decrease of MMSE, and predict MCI, mild AD, and moderate AD. It was the only significant predictor of severe AD. It differentiated the disease from HS at a sensitivity >90%, but exhibited only a poor–weak diagnostic power when the evaluation was carried out among the stages of AD. It should be noted that although 25(OH)D_3_ was decreased in MCI and AD, no difference was observed between women and men but it predicted a 2.5 times higher incidence of AD in women than in men. This result is in line with that reported 1.5–3 times higher incidence of AD in women than the incidence in men (Baum, 2005). Our results also showed that the decrease of 25(OH) D_3_ in patients with MCI and AD was not accompanied by a similar change of 1,25(OH)_2_D_3_; hence, serum 1,25(OH)_2_D_3_ concentration did not vary among HS and participants with MCI or AD. No change in 1, 25(OH)_2_D_3_ adds to the fact that concentration of 1,25(OH)_2_D_3_ is not a reliable marker in AD. Another finding of this study was that serum 1,25(OH)_2_D_3_ and 25(OH)D_3_ concentrations were not correlated in the HS group. It is noteworthy to mention that while 25(OH)D_3_ and 1,25(OH)_2_D_3_ concentrations were not correlated in men with AD, they were positively correlated in women patients with AD. These results may be associated with high incidence of AD in women and suggest gender differences. It could also result from possible limitation of 1,25(OH)_2_D_3_ synthesis from 25(OH)D_3_ in these patients. These results could suggest that vitamin D supplementation may be useful to patients with AD, especially in women, based on a positive correlation between serum 25(OH)D3 and 1,25(OH)2D3 in patients with AD.

## 5 CONCLUSION

MMSE and 25(OH)D_3_ are excellent–perfect predictors and diagnostic instruments for MCI and AD. The present study highlights the value of the combination of MMSE and 25(OH)D_3_ (but not 1,25(OH)_2_D_3_) as it provides an overall 98% prediction success rate. These results suggest that MMSE and 25(OH)D_3_ could support the clinical diagnosis of MCI and the mild, moderate, and severe stages of AD.

## CONFLICT OF INTEREST

The authors declare no conflicts of interests.
